# Tropical Fishes Dominate Temperate Reef Fish Communities within Western Japan

**DOI:** 10.1371/journal.pone.0081107

**Published:** 2013-12-03

**Authors:** Yohei Nakamura, David A. Feary, Masaru Kanda, Kosaku Yamaoka

**Affiliations:** 1 Graduate School of Kuroshio Science, Kochi University, Nankoku, Kochi, Japan; 2 School of the Environment, University of Technology, Sydney, New South Wales, Australia; 3 Kuroshio Zikkan Center, Otsuki, Kochi, Japan; Aristotle University of Thessaloniki, Greece

## Abstract

Climate change is resulting in rapid poleward shifts in the geographical distribution of tropical and subtropical fish species. We can expect that such range shifts are likely to be limited by species-specific resource requirements, with temperate rocky reefs potentially lacking a range of settlement substrates or specific dietary components important in structuring the settlement and success of tropical and subtropical fish species. We examined the importance of resource use in structuring the distribution patterns of range shifting tropical and subtropical fishes, comparing this with resident temperate fish species within western Japan (Tosa Bay); the abundance, diversity, size class, functional structure and latitudinal range of reef fishes utilizing both coral reef and adjacent rocky reef habitat were quantified over a 2 year period (2008–2010). This region has undergone rapid poleward expansion of reef-building corals in response to increasing coastal water temperatures, and forms one of the global hotspots for rapid coastal changes. Despite the temperate latitude surveyed (33°N, 133°E) the fish assemblage was both numerically, and in terms of richness, dominated by tropical fishes. Such tropical faunal dominance was apparent within both coral, and rocky reef habitats. The size structure of the assemblage suggested that a relatively large number of tropical species are overwintering within both coral and rocky habitats, with a subset of these species being potentially reproductively active. The relatively high abundance and richness of tropical species with obligate associations with live coral resources (i.e., obligate corallivores) shows that this region holds the most well developed temperate-located tropical fish fauna globally. We argue that future tropicalisation of the fish fauna in western Japan, associated with increasing coral habitat development and reported increasing shifts in coastal water temperatures, may have considerable positive economic impacts to the local tourism industry and bring qualitative changes to both local and regional fisheries resources.

## Introduction

The world's oceans have substantially warmed since 1955 [1], and there is increasing evidence that the geographic range of marine organisms has shifted in accordance with this warming [2]–[4]. One of the most well documented shifts in marine communities has been the poleward movement of habitat-forming species [3]. For example, within Japan over the last 30 years, tropical habitat-forming macroalgal *Sargassum* (Fucales) species have substantially increased in cover within temperate regions, while temperate *Sargassum* species have significantly decreased in cover [5]. In addition, in response to rising seawater temperatures, the range of four hermatypic (reef-building) coral species, including two common tropical *Acropora* species, have been expanding throughout temperate Japanese coasts since 1930, with the speed of these expansions reaching ∼14 km/year [6].

There is increasing evidence that tropical fish populations may be expanding into temperate regions, but there is still little information on the factors which may facilitate or constrain such expansion [4], [7], [8]. Tropical range shifts are likely to be limited by species-specific resource requirements [9], [10]. In particular, for tropical fishes, temperate reefs may lack a range of settlement substrates, settlement cues or specific tropical dietary components [11]. As approximately 10% of coral reef fishes are classified as coral dependent throughout some part of their life stage [12], for these species a reliance on live coral resources is likely to constrain shifts into temperate reef habitats [13]–[15]. For example, previous studies suggests that highly specialised trophic groups (e.g., obligate coral-feeding butterflyfishes) will be unlikely to recruit and survive in habitats devoid of extensive cover of preferred coral species [12], [16]. We can therefore expect then that the availability of specific coral reef resources may strongly constrain range extensions for coral reef fishes. Within regions devoid of high live coral cover, fish communities are expected to comprise species with little reliance on coral habitats and the resources they provide [4], independent of increased coastal temperatures [9].

**Figure 1:**
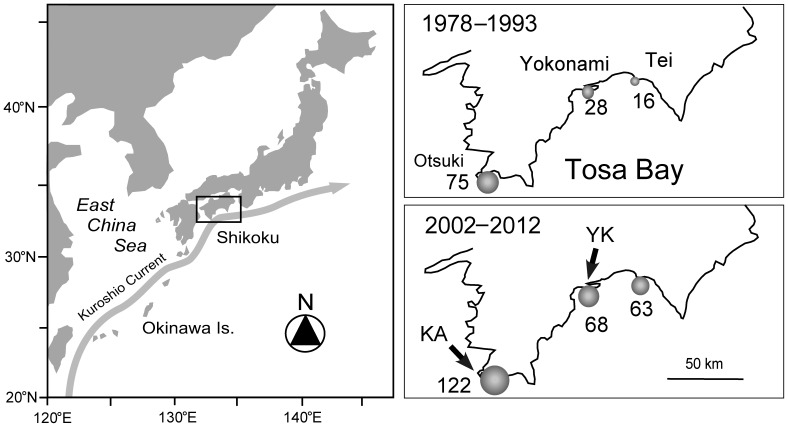
Map of the study site showing the location of Tosa Bay, western Japan. Figures on the right show the species richness of hermatypic corals observed at Otsuki, Yokonami, and Tei along the coast of Tosa Bay during the 1978–1993 and 2002–2012 survey periods, respectively. The numbers indicate coral species richness, and the gray circle indicates the degree of coral species richness. Arrows indicate the study sites (YK, Yokonami; KA, Kashiwajima). The coral species richness map was modified from that of Mezaki and Kubota [19].

**Figure 2:**
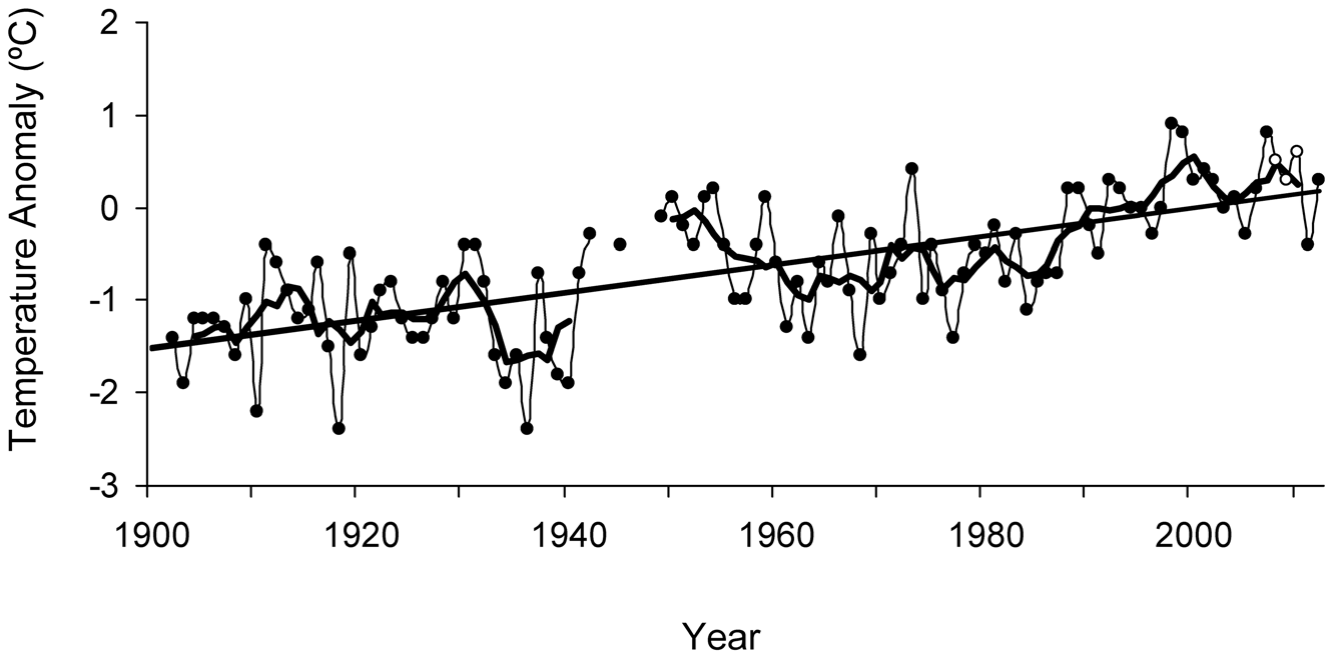
Time-series representation of winter surface seawater temperature annual anomalies at around offshore of Tosa Bay. Time series plot of annual averaged winter (Jan–Mar) surface seawater temperature anomalies (•) at around offshore of Tosa Bay for the period from 1902 to 2012. The 1981–2010 average is used as the normal. (○); study period (2008–2010). The bold line shows the five-year running mean, and the straight line indicates the long-term linear trend (*y* = 0.0151x−1.5287). Data were provided by the Japan Meteorological Agency.

There is increasing evidence for the expansion of reef-building corals into temperate regions globally, which has been closely associated with the proximity of regions to western boundary currents [6], [17], [18]. For example, Tosa Bay (western Japan, Fig. 1) has shown globally significant increases in coastal water temperatures and concomitant increases in coral development [19], [20]. This bay is strongly influenced by the offshore Kuroshio Current [20]. Winter sea surface temperature (SST) offshore of the bay is higher than average for Japanese temperate zones, rising approximately 1.51°C from 1902 to 2012 (Fig.2), double the average rate of global ocean warming [21]. In particular, mean winter SST (January - March) in central Tosa Bay has increased by approximately 1.7°C (from 15.8°C to 17.5°C) over the past 30 years [22]. With increased SSTs hermatypic coral habitats have been rapidly expanding within areas of the bay since the 1990s [19]. At present, 136 coral species belonging to 50 genera (16 families) have been surveyed in the bay [19], [20]. Such coral communities have shown extensive increases in species richness within the last 20 years (Fig. 1), with the tropical corals *Acropora muricata* and *A. latistella* having now developed into stable and permanent coral communities within the bay [19].

Although the expansion of tropical habitats (i.e., hermatypic corals and tropical algal beds) into temperate regions may be expected to facilitate the settlement and survival of tropical and subtropical fish species, for a range of resident temperate fish species such expansion may result in reduced cover of preferred temperate habitats, and concomitant reductions in the abundance of species closely associated with these habitats [22]. In addition, rapid changes in coastal water temperatures may also have a substantial mediating influence on species ability to compete for resources. For example, recent work (Beck et al. In review) suggests that competitive interactions between tropical vagrants and temperate residents are predominantly associated with temperature fluctuations; within warmer waters vagrants may numerically dominate fish populations, whereas with cooler waters such numerical dominance substantially reduces. Despite this, there is still little empirical evidence to determine the role of competition for resources in structuring patterns in tropical range shifts [4]. As space limitation is important in determining juvenile abundance in numerous site attached tropical fish species [23]–[26], we can expect that species specific differences in competition for temperate benthic resources both during and following settlement may have substantial consequences for the abundance and diversity of tropical and subtropical range-shifting species within temperate ecosystems.

The aim of this work was to determine the importance of tropical benthic habitats (i.e., hermatypic coral) and temperate rocky reef habitat in determining the abundance, diversity, size structure and functional composition of tropical and subtropical range shifting species, and temperate resident fish populations. Therefore, this work examined the composition of fish communities, within bimonthly surveys over 2 years, associated with adjacent coral – and rocky reef habitats within the temperate coastline of western Japan. We hypothesized that the abundance and diversity of tropical and subtropical fishes would be constrained by the availability of live coral reefs, correlated with species coral resource specialization. In parallel, the structure and function of existing temperate fish communities were expected to be substantially associated with temperate habitat availability, with little utilization of tropical habitats.

## Materials and Methods

### Ethics Statement

This study involved no capture or handling of fishes. This research was approved by local fisheries cooperative and the Kochi Prefecture.

### Study sites

**Figure 3:**
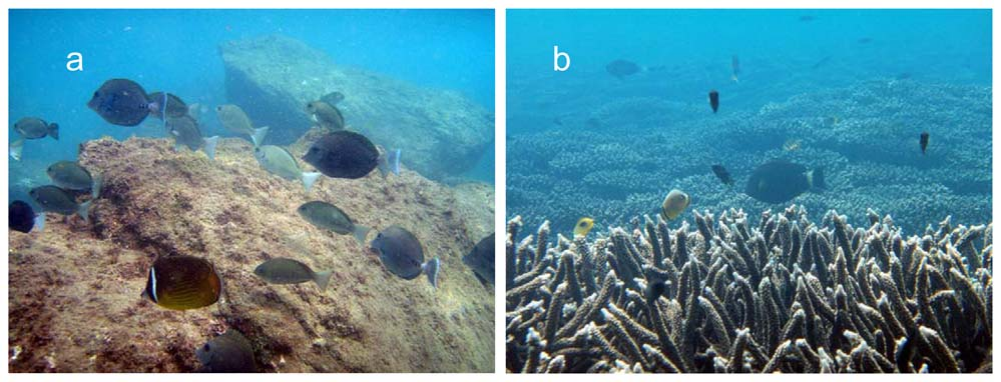
Underwater view of Yokonami, central Tosa Bay. (a) rocky and (b) coral habitats.

This study was conducted in Tosa Bay, western Japan (33°N, 133°E) (Fig. 1). Two locations were selected in Tosa Bay: the Yokonami Peninsula (YK) and Kashiwajima (KA) (Fig. 1). Within each location 2 separate study sites were designated according to the dominant benthic community composition: coral habitats were designated as areas where live coral cover exceeded >60% (at the transect level), whereas rocky reef sites were composed of 100% rock habitat (Fig. 3).

YK is a small peninsula located in central Tosa Bay, with both study sites located on the Pacific Ocean side of the peninsula. One site was dominated by a relatively shallow (2–8 m) and large (3 ha) coral reef, predominantly composed of branching and tabular *Acropora* (e.g., 50%–90% live coral cover dominated by *A. muricata*, *A. solitaryensis*, and *A. hyacinthus*, [27]). The second study site at YK was 100 m from the coral dominated site and encompassed 100% temperate rocky reef habitat (2–7 m depth, 4.5 ha). The second location (KA) is a small island (3.9 km circumference, with an area of 0.57 km^2^) located in Otsuki, the southwestern end of Tosa Bay. This location holds 122 coral species [19], with the coral-dominated site surveyed (within 2–10 m depth) holding patchily distributed coral reefs, dominated by tabular *Acropora* and *Pavona decussata* (>50% live coral cover); this sites was ∼100 m away from the rocky reef dominated site.

Temperature loggers (YK and Ohtoshima [15 km from KA, but having similar water temperature to KA]) deployed at 5 m recorded SST from Dec 1, 2008 to December 30, 2010, while salinity at each location was measured *in situ* using a portable salinometer in August and December each year.

### Survey protocols

Fish assemblages were surveyed bimonthly at each site using underwater visual belt transect surveys from December 2008 to December 2010 (52 surveys in total). During each bimonthly survey, five 20×1 m haphazardly laid transects, separated by at least 10 m, were utilised and all fishes within transects counted. All individuals were taxonomically identified to species (using [28]), and their total length estimated to 1 cm (roving fishes) and 0.5 cm (resident fishes). Fishes were categorized by latitudinal group (tropical, subtropical, and temperate) and primary feeding guild (i.e., benthivore, planktivore, corallivore, herbivore, omnivore, detritivore, piscivore, and parasite cleaner) based on FishBase [29]. All surveys were undertaken between 0900 and 1500 h.

### Data analysis

Fish species richness and abundance (20 m^2^) were compared between habitat types (coral reef, rocky reef; n = 5 in each habitat) and sampling months (total 13 months) within each location using mixed-model two-way analysis of variance (ANOVA). In this analysis, habitat type was considered to be a fixed factor and sampling month a random factor. Before analyses, all data were square root transformed (X+0.5). In addition, using separate mixed-model two-way analyses of variance, the abundance of fishes between latitudinal ranges and functional groups were examined between habitat types and sampling months within each location. Species richness and abundance were expressed per 20 m^2^. All statistical analyses were conducted using SPSS (14.0J).

## Results

### Fish assemblage structure

The majority of species surveyed within both coral and rocky habitats were tropical in origin (comprising 83.3% of total species diversity within each site), with relatively low species richness of subtropical or temperate fish species observed (comprising 10.1% and 6.4%: Table 1). In parallel the numerical abundance of individuals throughout both coral and rocky habitats was dominated by tropical fishes (85.6% of the total number of individuals surveyed over the 2 years), while both the subtropical fauna (9.7%) and temperate fauna (4.6%) held comparatively low abundances (Table S1)

**Table 1:** Species richness of each range group in rocky and coral habitats at Yokonami and Kashiwajima during the study period.

	Yokonami			Kashiwajima			
Range group	Rock	Coral	Total	Rock	Coral	Total	Total
Tropical fish	40	61	80	107	174	199	221
Subtropical fish	15	11	17	22	22	27	27
Temperate fish	5	4	5	11	14	16	17

**Figure 4:**
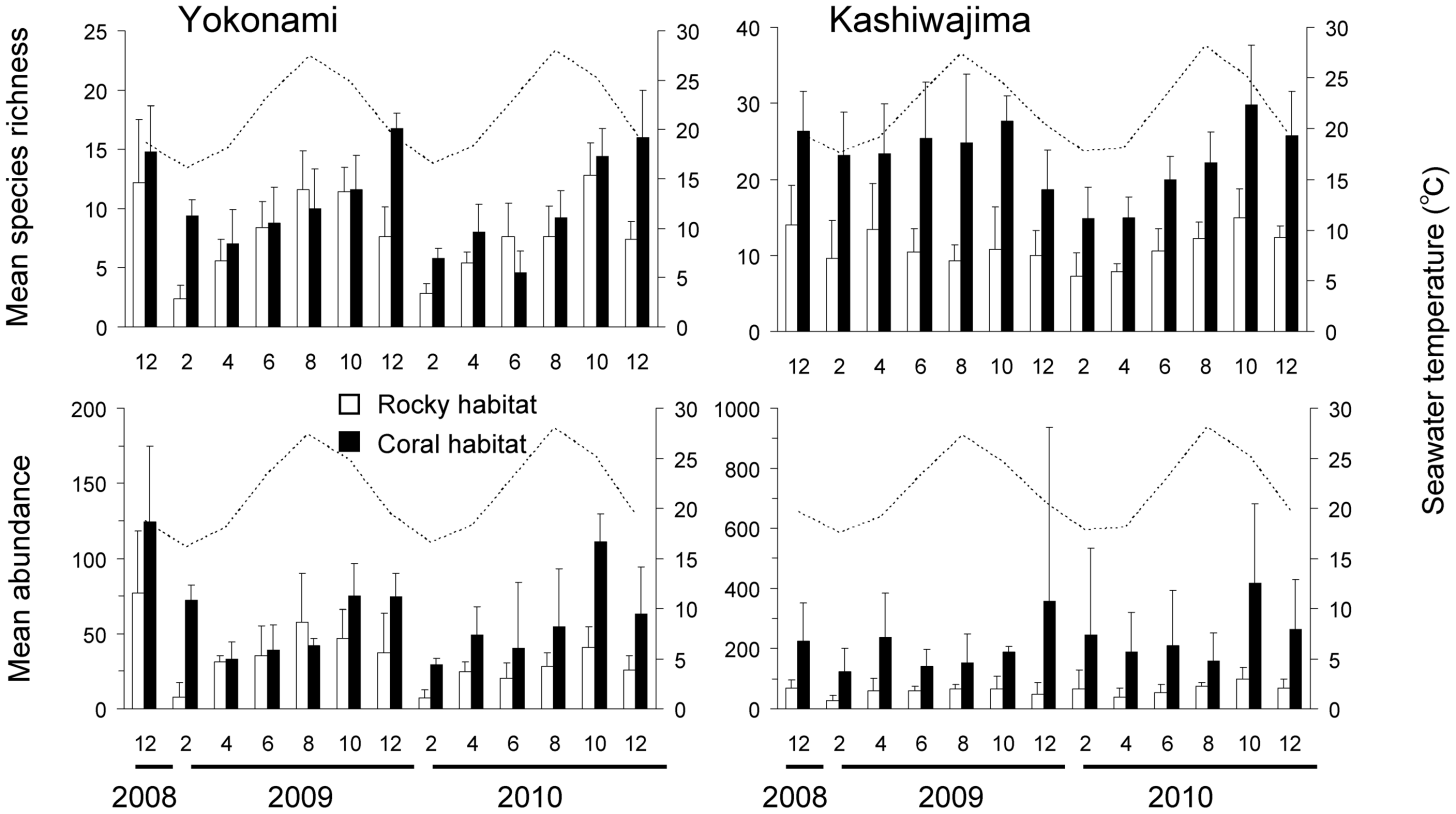
Seasonal patterns in species richness and abundance of fishes in the rocky and coral habitats. Bars indicate mean species richness and abundance [+standard deviation (SD)] of fishes per transect (n = 5, 1×20 m) in the rocky habitat and coral habitat. Months are labeled by numbers. The dotted line indicates the monthly average seawater temperature (depth, 5 m) around each site. Please note differences in y-axis scale between graphs.

**Figure 5:**
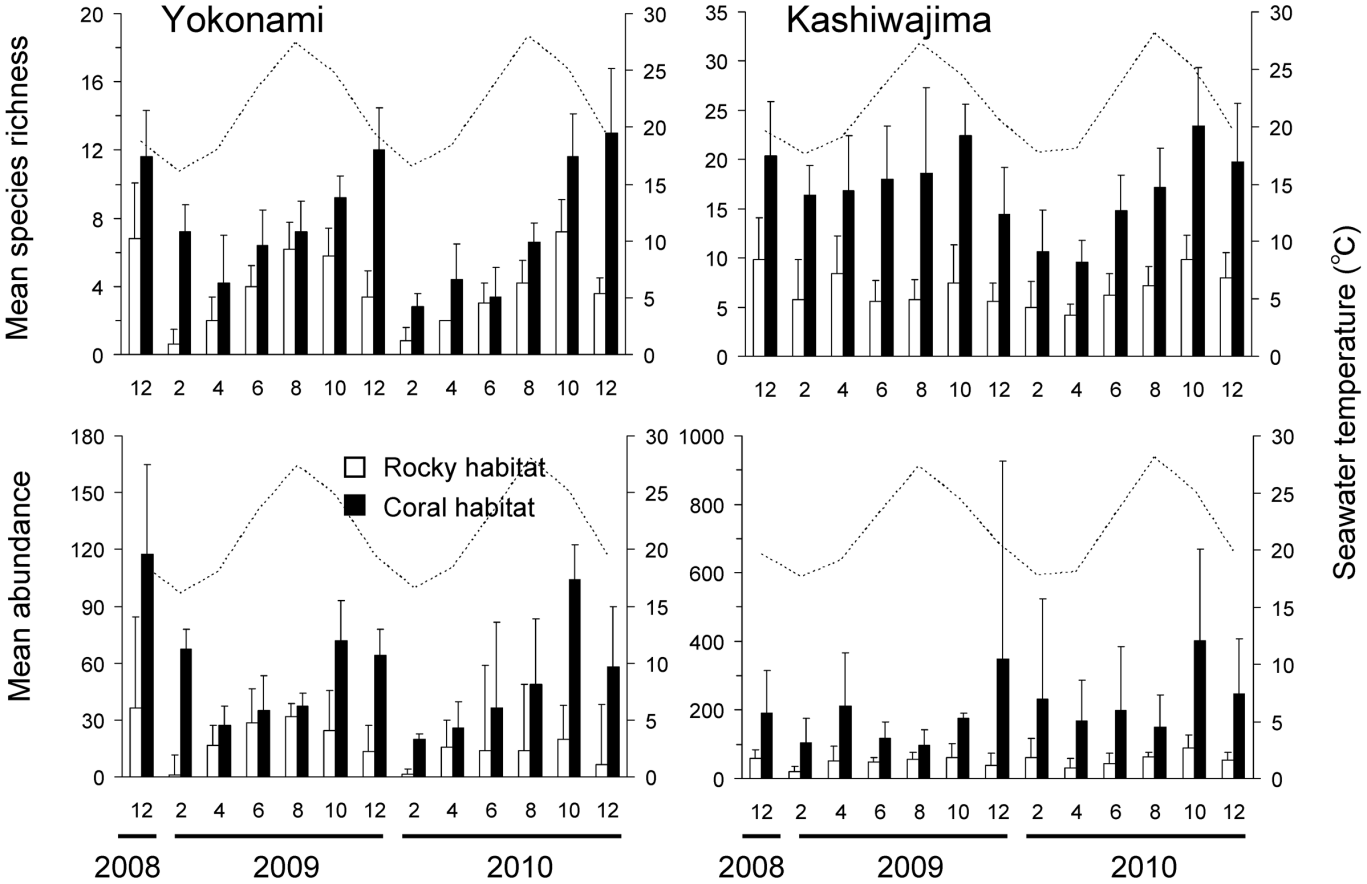
Seasonal patterns in species richness and abundance of tropical fishes in the rocky and coral habitats. Bars indicate mean species richness and abundance [+standard deviation (SD)] of fishes per transect (n = 5, 1×20 m) in the rocky habitat and coral habitat. Months are labeled by numbers. The dotted line indicates the monthly average seawater temperature (depth, 5 m) around each site. Please note differences in y-axis scale between graphs.

The coral habitat within both locations held significantly higher species richness and abundance than the rocky habitat (YK Species richness: *F*_1,104_ = 6.5, *p*<0.05, YK species abundance: *F*_1,104_ = 19.5, *p*<0.01; KA species richness: *F*_1,104_ = 267.4, *p*<0.01, species abundance: *F*_1,104_ = 154.5, *p*<0.01; Fig. 4). The high diversity and abundance of fishes within the coral habitat was primarily tropical in origin, with significantly higher tropical species richness and abundance in the coral than rocky habitat at both the locations (two-way ANOVA: YK, *F*_1,104_ = 28.5 and 31.1, *p*<0.001 for species richness and abundance; KA, *F*_1,104_ = 291.5 and 108.5, *p*<0.001 for species richness and abundance; Fig. 5). There were varying patterns in the richness and diversity of subtropical fauna between habitats at both locations. There was little difference in the richness or abundance of subtropical species between habitats at YK (two-way ANOVA: *F*_1,104_ = 3.6, *p* = 0.08 for species richness and *F*_1,104_ = 0.9, *p* = 0.36 for abundance), while within KA the coral habitat held significantly higher species richness and abundance of subtropical fishes than within the rocky habitat (two-way ANOVA: *F*_1,104_ = 52.7, *p*<0.001 for species richness and *F*_1,104_ = 21.4, *p*<0.01 for abundance). With YK there was significantly higher temperate faunal richness and abundance in the rocky habitat than the coral habitat (two-way ANOVA: *F*_1,104_ = 25.3, *p*<0.001 for species richness and *F*_1,104_ = 19.0, *p*<0.01 for abundance), whereas there were no significant differences in temperate species richness or abundance between habitats at KA (two-way ANOVA: *F*_1,104_ = 0.7, *p* = 0.43 for species richness and *F*_1,104_ = 0.2, *p* = 0.91 for abundance).

YK and KA species numbers within coral habitats was dominated by tropical butterflyfish (YK: 19.7%, KA: 10%) and tropical wrasses (YK: 11.8%, KA: 16.2%), while the species numbers associated with the rocky habitat were dominated by tropical damsels (15%) and subtropical wrasses (10%) at YK, and tropical wrasses (15%) at KA (Table S1). The numerical composition of fish assemblages at both locations within coral habitats were dominated by tropical damsels (YK: 30.1%, KA: 34.9%), while tropical butterflyfish (45.1%) in YK and tropical cardinalfish (41.1%) in KA were also numerically dominant in the coral habitats (Table S1). The fish communities within the rocky habitats at both locations were relatively dissimilar, with KA numerically dominated by tropical damsels (54.7%) and tropical cardinalfish (13%), while YK assemblages were numerically dominated by tropical damsels (37.9%), with lower abundances of subtropical wrasses (12.7%) and temperate wrasses (21.2%).

### Seasonal patterns in fish assemblage structure

There was a substantial decline in assemblage richness and abundance approximately 1–2 months following seasonal reductions in SST, with significantly more abundant and rich assemblages during middle boreal summer to early boreal winter (Aug to Dec), than middle boreal winter to early boreal summer (Feb to Jun) (two-way ANOVA: YK Species richness: *F*_12,104_ = 3.0, *p*<0.05, YK species abundance: *F*_12,104_ = 2.9, *p*<0.05; KA species richness: *F*_12,104_ = 6.1, *p*<0.01; KA species abundance: *F*_12,104_ = 3.0, *p*<0.05; Fig. 4).

There were clear differences in the effect of SST changes on fish assemblage structure, dependent on species latitudinal distribution range. Tropical species showed a clear increase and then decrease in both abundance and richness closely matched with seasonal changes in SST (two-way ANOVA: YK Species richness: *F*_12,104_ = 4.1, *p*<0.05, YK species abundance: *F*_12,104_ = 1.7, *p* = 0.18; KA species richness: *F*_12,104_ = 6.3, *p*<0.01, KA species abundance: *F*_12,104_ = 2.5, *p* = 0.06), (Fig. 5). In addition, within this fauna, despite a substantial decrease in abundance after December, individuals of 40 species were surveyed during the middle of winter (February) and following early spring (April) (Table S2). For several species there was also evidence to suggest that overwintering of breeding sized individuals within coral reef habitats is occurring (i.e., *Chaetodon auripes*: ≥12 cmTL, *Dascyllus reticulatus*: ≥6 cmTL, *Pomacentrus coelestis*: ≥6 cmTL, *P. nagasakiensis*: ≥8 cmTL ([30], Y Nakamura unpublished database) (Fig. S1). In comparison to the tropical fauna, there was no significant seasonal trend in both subtropical and temperate fishes richness or abundance (two-way ANOVA: *F*_12,104_ = 0.5–1.7, *p*>0.05 for species richness and abundance, excluding species richness of temperate fishes at KA [2-way ANOVA: *F*_12,104_ = 3.1, *p*<0.05]).

### Functional composition of fish assemblages

**Figure 6:**
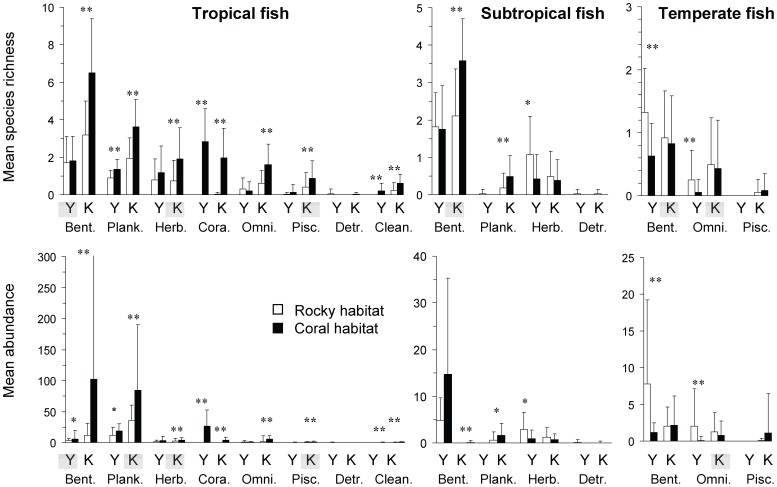
Functional composition of tropical, subtropical and temperate fish assemblages in the rocky and coral habitats. Bars indicate mean species richness and abundance [+standard deviation (SD)] of each feeding group of tropical, subtropical, and temperate fishes per transect in the rocky and coral habitats at Yokonami (Y) and Kashiwajima (K) during the study period (n = 65 in each habitat, 1×20 m). *p<0.05 and **p<0.01 between coral and rocky habitats by two-way analysis of variance (ANOVA) in abundance at each site. Shaded site symbols indicate significant seasonal differences at *p*<0.05 by two-way ANOVA in abundance. Bent. (Benthivore), Plank. (Planktivore), Herb. (Herbivore), Cora. (Corallivore), Omni. (Omnivore), Pisc. (Piscivore), Detr. (Detritivore), Clean. (Parasite cleaner).

The functional composition of both the subtropical and temperate fauna was a subset of the functional diversity found within the tropical fauna (Fig. 6). However, all three latitudinal groupings were dominated (both in mean richness and abundance) by benthivores, while planktivores were relatively abundant and diverse within the tropical fauna. Between habitats (coral and rocky reef) there were significant differences in functional groups between latitudinal groupings. Within the tropical fauna, the species richness and abundance of planktivores, corallivores, and parasite cleaners were significantly higher in the coral than rocky habitats at both the locations (two-way ANOVA: YK Species richness: *F*_1,104_ = 10.6, 58.1 and 15.6, *p*<0.01, YK species abundance: *F*_1,104_ = 5.6, 29.9 and 17.7, *p*<0.05; KA species richness: *F*_1,104_ = 73.3, 45.7 and 30.5, *p*<0.01, KA species abundance: *F*_1,104_ = 40.8, 29.9 and 25.0, *p*<0.01), while significantly higher abundances of benthivores, herbivores, omnivores, and piscivores were surveyed in the coral than rocky habitat in KA (two-way ANOVA: Species richness: *F*_1,104_ = 81.9, 91.0, 49.0 and 15.2, *p*<0.01, species abundance: *F*_1,104_ = 71.7, 21.8, 14.1 and 23.3, *p*<0.01; Fig. 6). Within the subtropical fauna both benthivores and planktivores showed significantly higher abundance and richness in the coral than rocky habitat at KA (two-way ANOVA: Species richness: *F*_1,104_ = 98.3 and 14.0, *p*<0.01, species abundance: *F*_1,104_ = 30.4 and 9.2, *p*<0.05), while a significantly higher abundance and richness of herbivores were found in the rocky habitat at YK (two-way ANOVA: Species richness: *F*_1,104_ = 8.0, *p*<0.05, species abundance: *F*_1,104_ = 6.2, *p*<0.05; Fig. 6). The temperate fauna showed significantly higher richness and abundance of benthivores and omnivores in the rocky, than coral habitat at YK (two-way ANOVA: Species richness: *F*_1,104_ = 20.1 and 16.8, *p*<0.01, species abundance: *F*_1,104_ = 14.4 and 11.7, *p*<0.01; Fig. 6).

### Environmental variables

There were distinct seasonal changes in average SST throughout both years of surveys, with the highest average SST across both locations within August (27.7°C±1.0), and the lowest average SST in February (17°C±1.2). However, both locations recorded SST lows of 15.3°C and 15.1°C in February, for KA and YK, respectively, while maximum SSTs of approximately 30°C (encompassing late August - early September) were recorded at both locations (Fig. 4). Salinity was similar at each location and season (approximately 34–35).

## Discussion

Despite both surveyed locations being temperate in climate (33°N, 133°E) the fish fauna was both numerically, and in terms of richness, dominated by fishes with a tropical origin. This is the first study worldwide to show that a temperate reef habitat is numerically dominated by a tropical fauna [4]. Within the Pacific coastline of Japan tropical fish juveniles are routinely observed throughout summer and autumn, most of these larvae presumably being transported from southern subtropical regions (e.g., Ryukyu Islands) by the northern flowing Kuroshio Current [31], [32]; historical records show that such vagrant incursions have been observed in western Japan from before the mid-20^th^ century [33], [34], with several cold tolerant tropical species (e.g. *Siganus fuscescens*, *Calotomus japonicu*s and *Kyphosus* sp.) already resident within western Japan centuries ago [35]. For example, within Kamohara's catalog [fish list based on a collection of fishes made in Kochi Prefecture (Tosa Bay) from the late 1920's to the early 1960's] [34], 153 out of 244 tropical and subtropical species identified in the present study (63%) have been reported from Tosa Bay (Table S1). However, the mean winter SST of Tosa Bay before the 1940's was below 16°C (Fig. 2), which crosses the minimum average winter temperature threshold for most tropical reef fish survival (16°C–18°C) [7], [36]. In comparison, the mean winter SST of Tosa Bay has substantially increased over the last 30 years, and has remained relatively high (∼17°C) since the late 1980's (Fig. 2) [21], [22]. Such high winter SSTs have been associated with a well-acknowledged and permanent development of tropical benthic species (i.e., hermatypic coral and *Sargassum* spp.) within western Japan [5], [6], [19]. Therefore, although data is lacking on the temporal development of tropical and subtropical reef fish populations within this region, we assume that an increase in the rate of larval incursion into Tosa Bay and the development of permanent fish populations have occurred in the same time frame as apparent for tropical benthic communities.

Range shifts in tropical and subtropical fishes into temperate regions are expected to be limited by species specific resource requirements [4], [9], [10]. In particular, we would expect that temperate reefs may lack the range of resources important in the development of non-native assemblages, including specific settlement substrates, settlement cues, or dietary components found on tropical and subtropical reefs [11]. However, the present work has shown that for some tropical and subtropical reef fish species the availability of live coral resources may not necessarily determine their distribution patterns between coral and rocky-dominated habitats. Such generalist habitat use is expected to be predominantly exhibited by species that can effectively utilise one of more resource (e.g., habitat, trophic) available within both the habitat types. For example, the vast majority of tropical species found in both the coral and rocky habitats had no strong association with coral trophic resources, with these fish's predominantly comprising benthivores (benthic invertebrate feeders) and herbivores (which included browsers and scrapers; Table S1); trophic resources appropriate for both functional groups were available throughout coral and rocky reef habitats. In comparison, there was a significantly higher abundance and diversity of obligate corallivores and obligate coral settlers/dwellers in the coral than rocky habitats at both locations; some of these species numerically dominated the tropical assemblage within sites (e.g. *Chaetodon speculum* and *C. lunulatus*). This pattern in assemblage structure suggests that the availability of coral resources may likely constrain the latitudinal shift of a range of tropical species that are obligately associated with coral reef resources [4], [37]. In fact, recent evidence has suggested that tropical species that are highly associated with live coral may show little success within temperate environments [38]. For example, the overwhelming majority of butterflyfishes surveyed over 12 years within south eastern Australia, where there has been little hermatypic coral shift into temperate environments, were non-coral or facultative coral feeders, despite obligate coral feeding butterflyfishes being relatively abundant in the southern Great Barrier Reef [39]. In Tosa Bay, although corallivorous butterflyfishes had been collected until the early 1960's (Table S1), the previous work has reported them as rare or very rare within assemblages [34]. Therefore, we argue that the expansion of hermatypic coral habitats coupled with increasing coastal SSTs over the last 30 years has led to a substantial shift in the species richness and abundance of coral-associated tropical fishes in this temperate region.

There is evidence to suggest that a relatively large number of tropical fish species survived throughout the two winter seasons in Tosa Bay, with a small subset of these species overwintering that are potentially reproductively active. The establishment of permanent (i.e., breeding) populations at high latitudes is a key indicator of successful geographic range shifts in tropical fishes [4], [40]. However, there has been little prior evidence of tropical fish species forming reproductively active populations in temperate regions ([4], [41] but see [42]). There is broad evidence to show that tropical fishes are able to effectively withstand water temperatures much lower than predominantly found in tropical latitudes (i.e., within temperate regions: [7], [36], [41]). Moreover, there is evidence to suggest that high latitude populations of tropical fishes may compensate for a shorter reproductive season and lower water temperature by producing gametes at higher rates than is the case at lower latitudes [43], [44]. However, within western Japan, rapid increases in coastal water temperatures associated with a strengthening of the Kuroshio Current [45] may have reduced the oceanographic variables constraining reproduction in several tropical fishes, resulting in viable breeding populations developing within this region. Therefore, although winter is still a key bottleneck for survival and population establishment of tropical fishes within western Japan [27], [46], we can predict that substantial changes in winter SST within regions associated with the increasing strength of the Kuroshio Current [45], amid fluctuations in natural warming cycles (associated with the Pacific Decadal Oscillation) may result in the successful breeding, and therefore establishment of permanent populations of tropical fish communities within western Japanese regions.

Changes in the structure and function of temperate fish communities within Japan, associated with rapid shifts in climate, are expected to have flow-on economic impacts to the local tourism industry and bring qualitative changes to local and regional fisheries resources. Scuba diving and glass-bottomed boat tourism attract large numbers of tourists and generate important tourism revenue for this region [20]. In Kashiwajima, for example, the scuba diving industry began in 1992, and now brings up to 10,000–15,000 Japanese and international divers per year to this area [47]. This industry contributes substantially to the local economy, both directly (e.g., through scuba diving packages) and indirectly (e.g., accommodation, food services and other local tourism ventures). With predictions of increasing warming occurring in this region [48], we can expect the cover and diversity of tropical coral reefs (and associated fish communities) to become more dominant. Such future ‘tropicalisation’ of Tosa Bay, and western Japan is expected to then increase the economic value of dive-based tourism within the region, associated with warmer summer waters and more tropical and subtropical dominated reef communities. In addition to local changes in reef-based tourism, shifts in the structure of fish communities within temperate Japan are expected to have substantial impacts on the fisheries sector. As this work found a large range of families important in tropical fisheries within both locations (including grouper (F. Serranidae), parrotfish (F. Scaridae), snapper (F. Lutjanidae), and emperor fish (F. Lethrinidae)), we may predict with increasing coastal waters that local and regional fishing productivity may hold increasing proportions of such tropical fish resources [8], [48], [49].

## Supporting Information

Figure S1 **Seasonal size distribution of the most abundant 10 possible overwintering species.** Total 10 transects in each month at Yokonami (YK) and Kashiwajima (KA). Months are labeled by numbers. The dotted line indicates the monthly average seawater temperature (depth, 5 m) around each location. For the most abundant 10 possible overwintering species, see Table S2.
(PDF)

Table S1 **Total abundance of each fish spceis across all the transects in rocky and coral habitats at Yokonami and Kashiwajima during the study period.** n = 65 in each habitat in each location. *Recorded in Kamohara (1964)[34].
(PDF)

Table S2 **List of possible overwintering tropical species; i.e. species observed in both middle winter (February) and following early spring (April).** Numbers indicate total abundance of each tropical spceis across all the transects in rocky and coral habitats at Yokonami and Kashiwajima during February and April in 2009 and 2010.
(PDF)
