# Supplementary figures and images for: Tropical Fishes Dominate Temperate Reef Fish Communities within Western Japan
Source: PLoS One. 2013 Dec 3;8(12):e81107. doi: 10.1371/journal.pone.0081107 (PMC3849258; doi:10.1371/journal.pone.0081107)

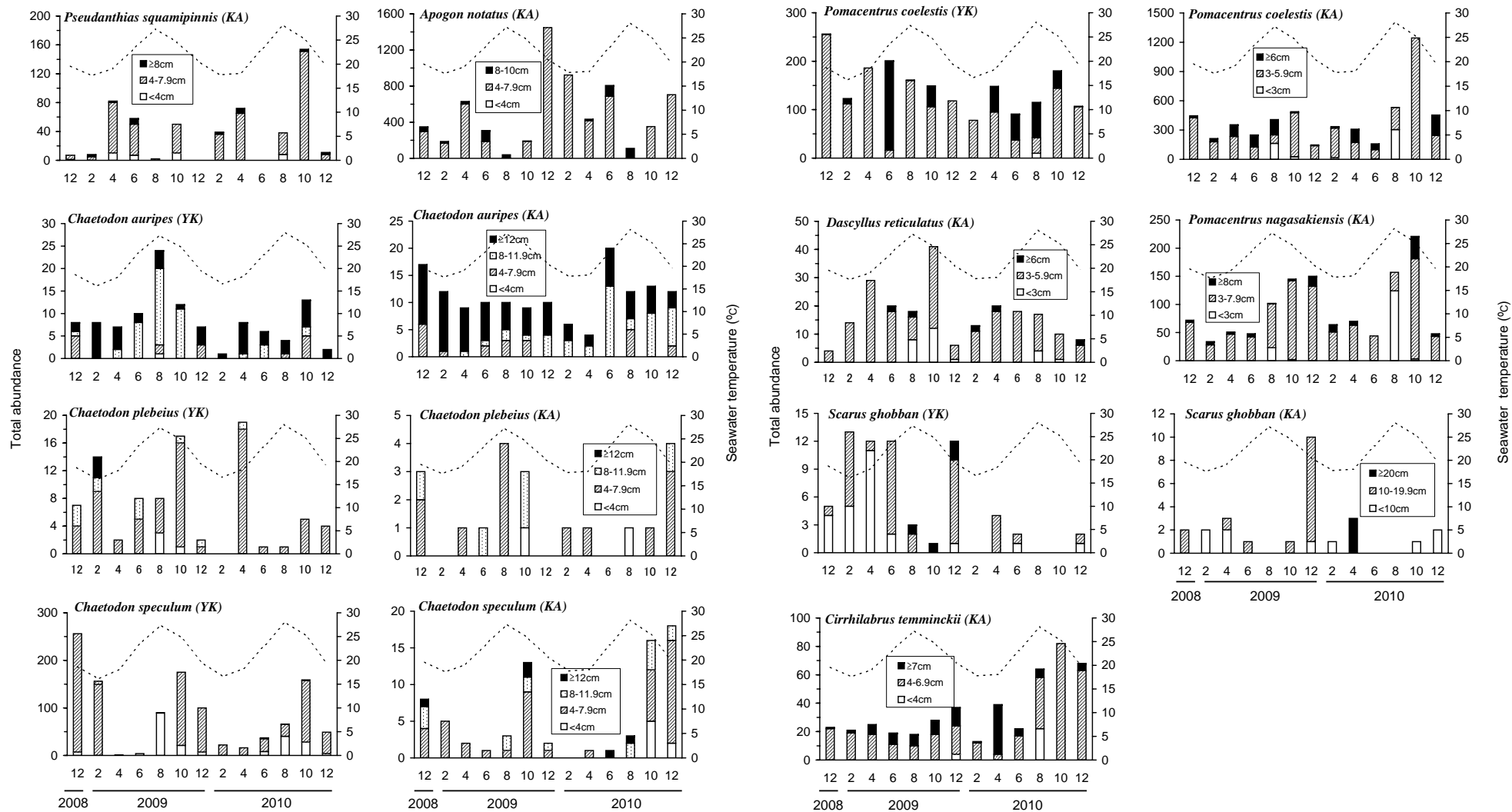

Supplement: Figure S1 — Seasonal size distribution of the most abundant 10 possible overwintering species. Total 10 transects in each month at Yokonami (YK) and Kashiwajima (KA). Months are labeled by numbers. The dotted line indicates the monthly average seawater temperature (depth, 5 m) around each location. For the most abundant 10 possible overwintering species, see Table S2. (PDF) [file pone.0081107.s001.pdf]
