# Supplementary material for: Tropical Fishes Dominate Temperate Reef Fish Communities within Western Japan
Source: PLoS One. 2013 Dec 3;8(12):e81107. doi: 10.1371/journal.pone.0081107 (PMC3849258; doi:10.1371/journal.pone.0081107)
Supplement: Table S1 — Total abundance of each fish spceis across all the transects in rocky and coral habitats at Yokonami and Kashiwajima during the study period. n = 65 in each habitat in each location. *Recorded in Kamohara (1964)[34]. (PDF) [file pone.0081107.s002.pdf]

Table S1. Total abundance of each fish spceis across all the transects in rocky and coral habitats at Yokonami and Kashiwajima during the study period.
n = 65 in each habitat in each location. *Recorded in Kamohara (1964)[34]

					Yokonami		Kashiwajima		
Family		Species	Distribution range	Feeding group	Rock	Coral	Rock	Coral	Total
Muraenidae		*Echidna nebulosa*	Tropical	Benthivore	0	0	0	1	1
	*	*Enchelycore pardalis*	Tropical	Piscivore	0	0	3	1	4
	*	*Gymnothorax kidako*	Tropical	Piscivore	0	0	1	4	5
	*	*Gymnothorax meleagris*	Tropical	Piscivore	0	0	0	2	2
	*	*Gymnothorax undulatus*	Tropical	Piscivore	0	0	0	1	1
Ophichthidae		*Myrichthys colubrinus*	Tropical	Benthivore	0	0	1	0	1
	*	*Myrichthys maculosus*	Tropical	Benthivore	0	0	0	1	1
Congridae	*	*Conger myriaster*	Temperate	Benthivore	0	0	0	1	1
Synodontidae		*Synodus dermatogenys*	Tropical	Piscivore	0	0	1	0	1
	*	*Synodus hoshinonis*	Tropical	Piscivore	0	0	1	0	1
		Synodus ulae	Tropical	Piscivore	0	0	1	0	1
	*	*Synodus variegatus*	Tropical	Piscivore	0	0	2	1	3
Gobiesocidae	*	*Diademichthys lineatus*	Tropical	Benthivore	0	0	34	84	118
Monocentridae	*	*Monocentris japonica*	Tropical	Benthivore	0	0	1	1	2
Holocentridae	*	*Myripristis berndti*	Tropical	Planktivore	0	0	0	3	3
	*	*Neoniphon sammara*	Tropical	Benthivore	0	0	1	1	2
	*	*Sargocentron ittodai*	Tropical	Benthivore	0	0	1	0	1
	*	*Sargocentron rubrum*	Subtropical	Benthivore	0	0	1	0	1
Aulostomidae	*	*Aulostomus chinensis*	Tropical	Piscivore	0	0	1	2	3
Fistulariidae	*	*Fistularia commersonii*	Tropical	Piscivore	0	1	3	10	14
Syngnathidae	*	*Corythoichthys haematopterus*	Tropical	Benthivore	0	0	8	4	12
Scorpaenidae		*Dendrochirus zebra*	Tropical	Piscivore	0	0	3	3	6
	*	*Pterois lunulata*	Tropical	Piscivore	0	0	2	8	10
	*	*Pterois volitans*	Tropical	Piscivore	0	0	0	3	3
		Scorpaena onaria	Temperate	Piscivore	0	0	1	0	1
	*	*Scorpaenodes littoralis*	Temperate	Piscivore	0	0	3	1	4
	*	*Sebastiscus marmoratus*	Tropical	Piscivore	1	6	1	11	19
	*	*Scorpaenopsis neglecta*	Tropical	Piscivore	0	0	1	0	1
Synanceiidae	*	*Inimicus japonicus*	Tropical	Piscivore	0	0	0	1	1
Serranidae	*	*Cephalopholis miniata*	Tropical	Piscivore	0	0	0	1	1
	*	*Epinephelus merra*	Tropical	Piscivore	0	0	0	1	1
	*	*Epinephelus fasciatus*	Tropical	Piscivore	0	0	3	10	13
	*	*Grammistes sexlineatus*	Tropical	Benthivore	0	0	1	1	2
	*	*Hyporthodus septemfasciatus*	Tropical	Piscivore	0	0	0	4	4
	*	*Plectropomus leopardus*	Tropical	Piscivore	0	2	0	1	3
	*	*Pseudanthias squamipinnis*	Tropical	Planktivore	0	0	38	483	521
Plesiopidae	*	*Plesiops coeruleolineatus*	Tropical	Benthivore	0	0	1	0	1
Apogonidae	*	*Apogon doederleini*	Subtropical	Benthivore	0	31	19	193	243
	*	*Apogon notatus*	Tropical	Benthivore	0	196	515	5966	6677
	*	*Apogon properuptus*	Subtropical	Benthivore	0	0	70	73	143
	*	*Cheilodipterus macrodon*	Tropical	Benthivore	3	0	5	21	29
		Cheilodipterus singapurensis	Tropical	Benthivore	0	0	3	6	9
Malacanthidae	*	*Malacanthus latovittatus*	Tropical	Benthivore	0	0	1	0	1
Scombropidae	*	*Scombrops boops*	Temperate	Benthivore	5	0	0	0	5
Carangidae	*	*Alectis ciliaris*	Subtropical	Benthivore	0	0	0	1	1
Lutjanidae	*	*Aprion virescens*	Tropical	Piscivore	0	0	3	2	5
	*	*Lutjanus gibbus*	Tropical	Benthivore	1	0	0	1	2
	*	*Lutjanus monostigma*	Tropical	Benthivore	0	0	0	20	20
	*	*Lutjanus russelli*	Tropical	Benthivore	0	1	0	0	1
	*	*Lutjanus stellatus*	Subtropical	Benthivore	2	1	3	0	6
Caesionidae	*	*Caesio teres*	Tropical	Planktivore	0	0	0	9	9
Gerreidae	*	*Gerres oyena*	Tropical	Benthivore	14	1	5	0	20
Haemulidae	*	*Diagramma pictum*	Tropical	Benthivore	1	2	0	1	4
	*	*Plectorhinchus picus*	Tropical	Benthivore	0	0	0	1	1
Nemipteridae		*Scolopsis bilineata*	Tropical	Benthivore	0	0	1	1	2
		Scolopsis lineata	Tropical	Benthivore	0	0	1	1	2
Sparidae	*	*Pagrus major*	Subtropical	Benthivore	1	0	1	0	2
Lethrinidae	*	*Lethrinus nebulosus*	Tropical	Benthivore	0	0	5	20	25
	*	*Lethrinus genivittatus*	Tropical	Benthivore	1	0	2	6	9
		Monotaxis grandoculis	Tropical	Benthivore	0	0	1	0	1
Mullidae		*Parupeneus barberinus*	Tropical	Benthivore	0	0	1	0	1
		Parupeneus ciliatus	Tropical	Benthivore	3	0	1	1	5
		Parupeneus cyclostomus	Tropical	Benthivore	0	0	1	0	1
	*	*Parupeneus indicus*	Tropical	Benthivore	1	2	2	1	6
	*	*Parupeneus multifasciatus*	Tropical	Benthivore	19	24	13	15	71
		Parupeneus pleurostigma	Tropical	Benthivore	0	0	1	1	2
	*	*Parupeneus spilurus*	Tropical	Benthivore	18	1	8	4	31
		Parupeneus trifasciatus	Tropical	Benthivore	1	0	0	0	1
		Mulloidichthys flavolineatus	Tropical	Benthivore	0	0	0	1	1
	*	*Mulloidichthys vanicolensis*	Tropical	Benthivore	0	0	11	0	11
	*	*Upeneus tragula*	Tropical	Benthivore	0	0	0	2	2
Kyphosidae	*	*Girella punctata*	Temperate	Omnivore	130	5	46	14	195
	*	*Kyphosus vaigiensis*	Tropical	Omnivore	1	0	0	0	1
	*	*Microcanthus strigatus*	Tropical	Omnivore	4	3	0	0	7
Ephippidae	*	*Platax teira*	Tropical	Benthivore	0	2	0	0	2
Chaetodontidae	*	*Chaetodon auripes*	Tropical	Benthivore	50	60	24	120	254
	*	*Chaetodon auriga*	Tropical	Benthivore	5	20	8	27	60
	*	*Chaetodon bennetti*	Tropical	Corallivore	0	3	0	1	4
		Chaetodon baronessa	Tropical	Corallivore	0	2	0	6	8
	*	*Chaetodon citrinellus*	Tropical	Benthivore	0	0	0	1	1
	*	*Chaetodon ephippium*	Tropical	Omnivore	0	1	0	0	1
	*	*Chaetodon kleini*	Tropical	Corallivore	0	0	1	4	5
	*	*Chaetodon lunula*	Tropical	Benthivore	0	0	1	2	3
	*	*Chaetodon lineolatus*	Tropical	Corallivore	0	1	0	1	2
	*	*Chaetodon melannotus*	Tropical	Corallivore	0	143	0	29	172
		Chaetodon oxycephalus	Tropical	Corallivore	0	7	0	6	13
	*	*Chaetodon plebeius*	Tropical	Corallivore	0	88	0	20	108
	*	*Chaetodon rafflesi*	Tropical	Benthivore	0	3	0	4	7
		Chaetodon semeion	Tropical	Omnivore	0	1	0	0	1

	*	_Chaetodon speculum_	Tropical	Corallivore	0	1131	0	73	1204
	*	_Chaetodon lunulatus_	Tropical	Corallivore	0	337	0	58	395
	*	_Chaetodon trifascialis_	Tropical	Corallivore	0	24	0	48	72
		Chaetodon ulietensis	Tropical	Omnivore	0	1	0	1	2
	*	_Chaetodon vagabundus_	Tropical	Omnivore	0	0	0	5	5
	*	_Coradion altivelis_	Tropical	Omnivore	0	0	0	4	4
	*	_Forcipiger flavissimus_	Tropical	Benthivore	0	0	0	1	1
		Heniochus monoceros	Tropical	Benthivore	0	0	0	5	5
		Heniochus varius	Tropical	Benthivore	0	0	0	3	3
Pomacanthidae		_Centropyge heraldi_	Tropical	Herbivore	0	0	0	1	1
	*	_Centropyge interruptus_	Temperate	Omnivore	0	0	0	3	3
	*	_Centropyge tibicen_	Tropical	Herbivore	0	0	9	53	62
		Centropyge vrolikii	Tropical	Herbivore	0	0	3	26	29
	*	_Chaetodontoplus septentrionalis_	Tropical	Benthivore	0	0	2	14	16
Oplegnathidae	*	_Oplegnathus fasciatus_	Temperate	Benthivore	0	0	2	0	2
	*	_Oplegnathus punctatus_	Tropical	Benthivore	0	0	3	0	3
Pomacentridae		_Abudefduf bengalensis_	Tropical	Omnivore	6	6	0	1	13
	*	_Abudefduf sexfasciatus_	Tropical	Planktivore	2	35	31	43	111
	*	_Abudefduf sordidus_	Tropical	Omnivore	2	0	0	0	2
	*	_Abudefduf vaigiensis_	Tropical	Omnivore	33	12	10	25	80
	*	_Amphiprion clarkii_	Tropical	Planktivore	0	23	5	76	104
		Amphiprion ocellaris	Tropical	Omnivore	0	1	0	0	1
	*	_Chromis analis_	Tropical	Planktivore	0	0	1	0	1
	*	_Chromis chrysura_	Tropical	Planktivore	0	0	0	12	12
	*	_Chromis flavomaculata_	Subtropical	Planktivore	0	0	0	2	2
	*	_Chromis fumea_	Tropical	Planktivore	0	0	0	5	5
		Chromis lepidolepis	Tropical	Planktivore	0	0	2	8	10
	*	_Chromis margaritifer_	Tropical	Planktivore	0	0	0	13	13
	*	_Chromis notata_	Subtropical	Planktivore	0	0	0	2	2
		Chromis weberi	Tropical	Planktivore	0	0	0	115	115
	*	_Chrysiptera cyanea_	Tropical	Omnivore	0	0	87	0	87
		Dascyllus reticulatus	Tropical	Omnivore	0	0	0	218	218
		Dascyllus trimaculatus	Tropical	Omnivore	0	0	12	44	56
		Neoglyphidodon melas	Tropical	Benthivore	0	0	3	0	3
	*	_Neoglyphidodon nigroris_	Tropical	Omnivore	0	0	0	1	1
	*	_Plectroglyphidodon leucozonus_	Tropical	Herbivore	9	0	0	1	10
		Plectroglyphidodon lacrymatus	Tropical	Omnivore	0	0	0	5	5
		Plectroglyphidodon johnstonianus	Tropical	Omnivore	0	0	0	1	1
		Plectroglyphidodon dickii	Tropical	Omnivore	0	0	0	3	3
	*	_Pomacentrus bankanensis_	Tropical	Omnivore	1	0	0	0	1
		Pomacentrus chrysurus	Tropical	Herbivore	1	0	0	0	1
	*	_Pomacentrus coelestis_	Tropical	Planktivore	777	1136	1606	3734	7253
	*	_Pomacentrus nagasakiensis_	Tropical	Planktivore	0	1	439	767	1207
		Pomachromis richardsoni	Tropical	Benthivore	0	0	0	4	4
	*	_Stegastes altus_	Subtropical	Herbivore	30	2	0	7	39
		Stegastes fasciolatus	Tropical	Detritivore	6	0	0	0	6
Cirrhitidae	*	_Cirrhitichthys aprinus_	Tropical	Benthivore	0	0	0	3	3
		Paracirrhites arcatus	Tropical	Benthivore	0	0	0	18	18
	*	_Cirrhitichthys aureus_	Tropical	Benthivore	0	0	0	1	1
		Cirrhitichthys falco	Tropical	Benthivore	0	0	0	3	3
	*	_Paracirrhites forsteri_	Tropical	Benthivore	0	0	0	15	15
Cheilodactylidae	*	_Goniistius zonatus_	Tropical	Benthivore	34	1	9	4	48
	*	_Goniistius zebra_	Temperate	Benthivore	0	0	1	2	3
Mugilidae	*	_Mugil cephalus_	Subtropical	Detritivore	5	0	3	0	8
Labridae	*	_Anampses caeruleopunctatus_	Tropical	Benthivore	0	0	2	3	5
		Anampses melanurus	Tropical	Benthivore	0	0	0	1	1
	*	_Anampses meleagrides_	Tropical	Benthivore	0	0	0	11	11
		Anampses twistii	Tropical	Benthivore	0	0	1	2	3
		Bodianus axillaris	Tropical	Benthivore	0	0	0	2	2
	*	_Bodianus bilunulatus_	Tropical	Benthivore	0	0	0	1	1
		Bodianus dictynna	Tropical	Benthivore	0	0	0	1	1
	*	_Cheilio inermis_	Tropical	Benthivore	1	0	0	0	1
		Cheilinus celebicus	Tropical	Benthivore	0	0	0	2	2
		Cheilinus chlorourus	Tropical	Benthivore	0	0	1	0	1
	*	_Choerodon azurio_	Subtropical	Benthivore	0	0	1	1	2
	*	_Cirrhilabrus temminckii_	Tropical	Planktivore	0	0	164	295	459
	*	_Coris aygula_	Tropical	Benthivore	0	0	1	1	2
	*	_Coris batuensis_	Tropical	Benthivore	0	1	0	2	3
	*	_Coris dorsomacula_	Tropical	Benthivore	0	0	5	11	16
	*	_Coris gaimard_	Tropical	Benthivore	0	0	0	3	3
		Cymolutes torquatus	Tropical	Benthivore	0	0	3	3	6
	*	_Gomphosus varius_	Tropical	Benthivore	0	7	5	31	43
		Halichoeres chrysus	Tropical	Benthivore	0	0	5	12	17
		Halichoeres hortulanus	Tropical	Benthivore	0	0	0	1	1
	*	_Halichoeres marginatus_	Tropical	Benthivore	0	0	2	1	3
		Halichoeres melanurus	Tropical	Benthivore	0	0	0	5	5
	*	_Halichoeres melanochir_	Tropical	Benthivore	2	0	4	5	11
		Halichoeres nebulosus	Subtropical	Benthivore	5	0	5	0	10
		Halichoeres prosopeion	Tropical	Benthivore	0	0	1	3	4
		Halichoeres scapularis	Tropical	Benthivore	0	0	1	0	1
		Halichoeres trimaculatus	Tropical	Benthivore	0	0	0	1	1
	*	_Halichoeres tenuispinis_	Tropical	Benthivore	38	26	12	13	89
	*	_Hemigymnus fasciatus_	Tropical	Benthivore	0	2	2	10	14
		Hemigymnus melapterus	Tropical	Benthivore	0	2	0	6	8
	*	_Hologymnosus annulatus_	Tropical	Benthivore	0	0	2	5	7
	*	_Labroides dimidiatus_	Tropical	Cleaner fish	0	15	19	55	89
	*	_Macropharyngodon meleagris_	Tropical	Benthivore	0	0	4	1	5
	*	_Oxycheilinus bimaculatus_	Tropical	Benthivore	0	0	4	3	7
	*	_Pseudolabrus eoethinus_	Subtropical	Benthivore	71	24	13	43	151
		Pseudolabrus sieboldi	Subtropical	Benthivore	0	0	1	2	3
		Pseudocheilinus hexataenia	Tropical	Benthivore	0	0	1	30	31
	*	_Stethojulis bandanensis_	Tropical	Benthivore	0	0	0	3	3
	*	_Stethojulis interrupta_	Temperate	Benthivore	467	75	100	124	766
		Stethojulis strigiventer	Tropical	Benthivore	0	1	0	0	1
	*	_Thalassoma amblycephalum_	Subtropical	Planktivore	4	0	38	103	145
	*	_Thalassoma cupido_	Subtropical	Benthivore	178	216	70	275	739
	*	_Thalassoma hardwicke_	Tropical	Benthivore	0	10	12	27	49
	*	_Thalassoma lutescens_	Subtropical	Benthivore	8	7	25	82	122

	*	*Thalassoma lunare*	Subtropical	Benthivore	14	54	80	246	394
	*	*Thalassoma purpureum*	Tropical	Benthivore	0	0	0	1	1
		Labridae spp.(unidentified juveniles)	Tropical	Benthivore	0	3	0	1	4
Scaridae	*	*Calotomus japonicus*	Subtropical	Herbivore	49	4	29	17	99
		Chlorurus microrhinos	Tropical	Herbivore	0	0	1	0	1
		Chlorurus sordidus	Tropical	Herbivore	0	1	3	6	10
		Scarus altipinnis	Tropical	Herbivore	0	2	0	3	5
		Scarus frenatus	Tropical	Herbivore	0	0	0	1	1
	*	*Scarus ghobban*	Tropical	Herbivore	11	55	2	24	92
	*	*Scarus ovifrons*	Subtropical	Herbivore	1	6	5	5	17
		Scarus prasiognathos	Tropical	Herbivore	0	3	0	0	3
		Scarus niger	Tropical	Herbivore	0	0	0	4	4
		Scarus rivulatus	Tropical	Herbivore	0	0	2	1	3
		Scarus rubroviolaceus	Tropical	Herbivore	0	0	1	2	3
		Scaridae sp.1	Tropical	Herbivore	0	1	0	0	1
		Scaridae spp.(unidentified juveniles)	Tropical	Herbivore	4	23	56	66	149
Pinguipedidae		*Parapercis kamoharai*	Subtropical	Benthivore	0	0	4	2	6
		Parapercis millepunctata	Tropical	Benthivore	0	0	0	1	1
	*	*Parapercis snyderi*	Subtropical	Benthivore	0	0	4	2	6
	*	*Parapercis tetracantha*	Tropical	Benthivore	0	0	1	0	1
Bleniidae		*Atrosalarias holomelas*	Tropical	Detritivore	0	0	0	1	1
		Ecsenius bicolor	Tropical	Herbivore	0	0	0	1	1
	*	*Istiblennius edentulus*	Tropical	Herbivore	1	0	0	0	1
	*	*Meiacanthus kamoharai*	Temperate	Omnivore	0	0	10	12	22
	*	*Plagiotremus tapeinosoma*	Tropical	Piscivore	0	0	1	0	1
	*	*Petroscirtes breviceps*	Tropical	Benthivore	0	0	5	4	9
	*	*Plagiotremus rhinorhynchos*	Tropical	Benthivore	0	0	0	1	1
Callionymidae		*Minysynchiropus kiyoae*	Temperate	Benthivore	0	0	0	1	1
		Neosynchiropus moyeri	Temperate	Benthivore	0	0	9	1	10
		Callionymidae sp.1	Temperate	Benthivore	0	0	0	1	1
Gobiidae		*Fusigobius neophytus*	Tropical	Benthivore	0	0	0	1	1
		Fusigobius duospilus	Tropical	Benthivore	0	0	0	1	1
		Gobiodon quinquestrigatus	Tropical	Corallivore	0	0	0	6	6
		Istigobius ornatus	Tropical	Benthivore	0	0	6	1	7
		Istigobius campbelli	Tropical	Benthivore	0	0	1	3	4
		Paragobiodon modestus	Tropical	Corallivore	0	0	0	2	2
		Ptereleotris evides	Tropical	Planktivore	0	11	10	26	47
	*	*Gobiodon* sp.1	Tropical	Corallivore	0	0	0	10	10
		Gobiodon sp.2	Tropical	Corallivore	0	0	0	9	9
		Eviota sp.	Tropical	Benthivore	0	0	0	1	1
Microdesmidae		*Gunnellichthys monostigma*	Tropical	Benthivore	0	2	0	9	11
Siganidae	*	*Siganus fuscescens*	Tropical	Herbivore	5	0	1	0	6
		Siganus spinus	Tropical	Herbivore	1	0	0	0	1
Zanclidae	*	*Zanclus cornutus*	Subtropical	Benthivore	0	3	5	18	26
Acanthuridae		*Acanthurus lineatus*	Tropical	Herbivore	3	3	0	0	6
	*	*Acanthurus dussumieri*	Tropical	Herbivore	48	73	18	13	152
	*	*Acanthurus nigrofuscus*	Tropical	Herbivore	3	26	36	10	75
		Acanthurus maculiceps	Tropical	Herbivore	3	0	0	0	3
		Acanthurus mata	Tropical	Planktivore	0	0	0	5	5
	*	*Acanthurus xanthopterus*	Tropical	Herbivore	0	0	0	7	7
		Ctenochaetus striatus	Tropical	Herbivore	0	0	0	1	1
		Naso lituratus	Tropical	Herbivore	0	1	0	2	3
	*	*Naso unicornis*	Tropical	Herbivore	5	5	0	1	11
	*	*Prionurus scalprum*	Subtropical	Herbivore	107	46	44	16	213
		Zebrasoma scopas	Tropical	Herbivore	0	2	0	3	5
	*	*Zebrasoma velifer*	Tropical	Herbivore	0	8	1	9	18
Sphyraenidae	*	*Sphyraena japonica*	Temperate	Piscivore	0	0	0	72	72
Balistidae	*	*Balistapus undulatus*	Tropical	Omnivore	0	0	0	2	2
	*	*Melichthys vidua*	Tropical	Herbivore	0	0	0	1	1
		Odonus niger	Tropical	Planktivore	0	0	1	1	2
	*	*Pseudobalistes flavimarginatus*	Tropical	Benthivore	0	1	0	0	1
	*	*Sufflamen chrysopterum*	Tropical	Benthivore	0	0	12	24	36
Monacanthidae	*	*Cantherhines dumerilii*	Tropical	Benthivore	0	0	1	1	2
		Paraluteres prionurus	Tropical	Benthivore	0	0	1	0	1
		Pervagor melanocephalus	Tropical	Benthivore	0	0	0	1	1
	*	*Rudarius ercodes*	Temperate	Benthivore	0	0	2	1	3
	*	*Stephanolepis cirrhifer*	Temperate	Omnivore	3	1	23	22	49
		Oxymonacanthus longirostris	Tropical	Corallivore	0	1	0	0	1
Ostraciidae	*	*Lactoria diaphana*	Subtropical	Benthivore	1	0	7	7	15
		Lactoria fornasini	Tropical	Benthivore	0	0	3	4	7
		Ostracion cubicus	Tropical	Omnivore	1	0	1	4	6
	*	*Ostracion immaculatus*	Temperate	Benthivore	4	1	8	6	19
Tetraodontidae	*	*Arothron hispidus*	Tropical	Omnivore	0	0	1	0	1
		Canthigaster coronata	Tropical	Omnivore	0	0	2	2	4
	*	*Canthigaster rivulata*	Tropical	Omnivore	1	0	3	2	6
	*	*Canthigaster valentini*	Tropical	Omnivore	0	0	7	10	17
Diodontidae	*	*Chilomycterus reticulatus*	Subtropical	Benthivore	0	0	0	1	1
	*	*Diodon holocanthus*	Subtropical	Benthivore	2	0	6	12	20
		Total species numbers			60	76	140	210	265
		Total individual numbers			2208	4039	4012	14566	24825
