# Supplementary material for: Tropical Fishes Dominate Temperate Reef Fish Communities within Western Japan
Source: PLoS One. 2013 Dec 3;8(12):e81107. doi: 10.1371/journal.pone.0081107 (PMC3849258; doi:10.1371/journal.pone.0081107)
Supplement: Table S2 — List of possible overwintering tropical species; i.e. species observed in both middle winter (February) and following early spring (April). Numbers indicate total abundance of each tropical spceis across all the transects in rocky and coral habitats at Yokonami and Kashiwajima during February and April in 2009 and 2010. (PDF) [file pone.0081107.s003.pdf]

Table S2. List of possible overwintering tropical species; i.e. species observed in both middle winter (February) and following early spring (April). Numbers indicate total abundance of each tropical spceis across all the transects in rocky and coral habitats at Yokonami and Kashiwajima during February and April in 2009 and 2010.

		Yokonami		Kashiwajima	
Family	Species	February	April	February	April
Gobiesocidae	*Diademichthys lineatus*	0	0	13	21
Fistulariidae	*Fistularia commersonii*	0	0	7	1
Serranidae	*Pseudanthias squamipinnis*	0	0	47	154
Apogonidae	*Apogon notatus*	0	0	1109	1062
	Cheilodipterus macrodon	0	0	3	1
	Cheilodipterus singapurensis	0	0	4	1
Caesionidae	*Caesio teres*	0	0	5	3
Gerreidae	*Gerres oyena*	0	0	2	2
Mullidae	*Parupeneus multifasciatus*	0	0	3	2
Chaetodontidae	*Chaetodon auripes*	9	15	18	13
	Chaetodon auriga	0	0	1	2
	Chaetodon plebeius	14	21	1	2
	Chaetodon speculum	178	17	5	3
	Chaetodon lunulatus	0	0	2	3
Pomacanthidae	*Centropyge tibicen*	0	0	9	8
	Centropyge vrolikii	0	0	3	1
	Chaetodontoplus septentrionalis	0	0	2	2
Pomacentridae	*Abudefduf vaigiensis*	2	1	1	0
	Amphiprion clarkii	1	3	8	10
	Dascyllus reticulatus	0	0	27	49
	Dascyllus trimaculatus	0	0	9	2
	Plectroglyphidodon dickii	0	0	1	1
	Pomacentrus coelestis	201	334	548	663
	Pomacentrus nagasakiensis	0	0	98	121
Cirrhitidae	*Paracirrhites forsteri*	0	0	1	1
Cheilodactylidae	*Goniistius zonatus*	2	2	4	3
Labridae	*Anampses meleagrides*	0	0	3	3
	Cirrhilabrus temminckii	0	0	34	64
	Coris dorsomacula	0	0	4	5
	Gomphosus varius	0	0	3	3
	Halichoeres tenuispinis	1	1	5	5
	Hemigymnus fasciatus	0	0	1	1
	Labroides dimidiatus	0	0	12	15
	Pseudocheilinus hexataenia	0	0	6	1
	Thalassoma hardwicke	0	0	7	3
Scaridae	*Scarus ghobban*	13	16	3	6
Microdesmidae	*Gunnellichthys monostigma*	0	0	2	1
Acanthuridae	*Acanthurus dussumieri*	7	9	0	0
Balistidae	*Sufflamen chrysopterum*	0	0	6	5
Tetraodontidae	*Canthigaster valentini*	0	0	4	2
